# Tremors in white rhinoceroses (*Ceratotherium simum*) during etorphine–azaperone immobilisation

**DOI:** 10.4102/jsava.v88i0.1466

**Published:** 2017-02-24

**Authors:** Stephanie S. de Lange, Andrea Fuller, Anna Haw, Markus Hofmeyr, Peter Buss, Michele Miller, Leith C.R. Meyer

**Affiliations:** 1 Department of Paraclinical Sciences, University of Pretoria, South Africa; 2 School of Physiology, University of the Witwatersrand, South Africa; 3 Veterinary Wildlife Services, South African National Parks, Kruger National Park, South Africa; 4 Department of Production Animal Sciences, University of Pretoria, South Africa; 5 Division of Molecular Biology and Human Genetics, Stellenbosch University, South Africa

## Abstract

Little is known about the mechanisms causing tremors during immobilisation of rhinoceros and whether cardiorespiratory supportive interventions alter their intensity. Therefore, we set out to determine the possible mechanisms that lead to muscle tremors and ascertain whether cardiorespiratory supportive interventions affect tremor intensity. We studied tremors and physiological responses during etorphine–azaperone immobilisation in eight boma-held and 14 free-living white rhinoceroses. Repeated measures analysis of variance and a Friedman test were used to determine differences in variables over time and between interventions. Spearman and Pearson correlations were used to test for associations between variables. Tremor intensity measured objectively by activity loggers correlated well (*p* < 0.0001; *r*^2^ = 0.9) with visual observations. Tremor intensity was greatest when animals were severely hypoxaemic and acidaemic. Tremor intensity correlated strongly and negatively with partial pressure of oxygen (PaO_2_) (*p* = 0.0003; *r*^2^ = 0.9995) and potential of hydrogen (pH) (*p* = 0.02, *r*^2^ = 0.97). It correlated strongly and positively with adrenaline concentrations (*p* = 0.003; *r*^2^ = 0.96), and adrenaline correlated strongly and negatively with PaO_2_ (*p* = 0.03; *r*^2^ = 0.95) and pH (*p* = 0.03; *r*^2^ = 0.94). Therefore, hypoxaemia and acidaemia were likely associated with the intensity of tremors through their activation of the release of tremorgenic levels of adrenaline. Tremors can be reduced if circulating adrenaline is reduced, and this can be achieved by the administration of butorphanol plus oxygen insufflation. Furthermore, to assist with reducing the risks associated with rhinoceros immobilisation, tremor intensity could be used as a clinical indicator of respiratory and metabolic compromise.

## Introduction

Management and conservation of the white rhinoceros necessitates the use of potent opioids such as etorphine, thiafentanil, fentanyl and carfentanil for immobilisation (Kock et al. 1995; Radcliffe & Morkel 2014). Opioids induce immobilisation by directly activating neurons in the brain to cause catalepsy and catatonia (Kania 1985). Unfortunately, during immobilisation opioids produce well-known side-effects, including muscle tremors, tachycardia, hypertension and respiratory depression (with subsequent hypoxaemia, hypercapnia and acidaemia), which may result in life-threatening complications (Atkinson et al. 2002; Buss et al. 2015, 2016; Fahlman 2008; Moreira 2010; Portas 2004; Radcliff, Ferrell & Childs 2000). Of these side-effects, the least studied is muscle tremors. Whether these tremors arise as a direct result of opioid receptor activation, as has been suggested in humans with opioid-induced myoclonus (Mercadante 1998; Vella-Brincat & Macleod 2007), or indirectly as a consequence of other physiological alterations in the immobilised rhinoceros, or both, is not yet known. The potent opioids used for chemical immobilisation have been implicated as a direct cause of muscle tremors in several different species (Burroughs et al. 2012b; Moreira 2010) and, in white rhinoceroses, tremors have been found to increase with additional doses of etorphine (Heard, Olsen & Stover 1992; Kock et al. 1995).

The tremors that are observed during chemical immobilisation of white rhinoceroses are likely to be rest tremors, which occur when the muscles are involuntarily stimulated (Findley 1996). These tremors usually present as a conspicuous shaking and trembling, sometimes making it difficult to work with an immobilised animal and potentially exacerbating the pathophysiological effects of capture and immobilisation. In an attempt to reduce tremors and other side-effects of opioids, sedatives and tranquillisers have been administered in combination with potent opioids (Mentaberre et al. 2010; Moreira 2010). Benzodiazepines and α_2_-agonists, sedatives that are commonly used during capture, have muscle-relaxant effects (Burroughs, Meltzer & Morkel 2012a). Although their effects on tremors have been clinically observed (Kock et al. 1995; Langhout et al. 2016; Moreira 2010; Wenger et al. 2007), these effects have not been adequately studied or described. Butorphanol, an opioid agonist–antagonist which has primarily antagonistic effects at µ-opioid receptors and agonist effects at κ-opioid receptors (Prado et al. 2008; Radcliffe et al. 2000), is used to reduce the cardiorespiratory side-effects of opioid-induced immobilisation. When butorphanol is administered with etorphine in a dart (Wenger et al. 2007), or administered intravenously to etorphine-immobilised white rhinoceroses (Burroughs et al. 2012b; Miller et al. 2013), it is reported to reduce tremors. Whether butorphanol alters muscle tremors directly through its effects on opioid receptors or indirectly by improving the animal’s cardiorespiratory function (Haw et al. 2014) needs to be clarified.

A captured animal’s physiology is likely to be altered not only by the immobilising drugs but also as a consequence of increased sympathetic nervous system activity associated with a ‘fight or flight’ response. Catecholamines, which are released into the circulation (Meltzer & Kock 2012; Moreira 2010), can be tremorgenic (Lakie 2010). Increased catecholamine concentrations can also lead to other effects, such as a decrease in arterial blood oxygen partial pressures and alterations in concentrations of blood glucose, potassium and potential of hydrogen (pH) (Grayson & Oyebola 1983; Moratinos & Reverte 1993; Primmett et al. 1986). Conversely, hypoxia and acidosis can stimulate release of catecholamines (Jeffers 1986; Nahas 1970; Perry et al. 1989; Rose, Purdue & Hensley 1977; Wasser & Jackson 1991; Yates et al. 2012). Tremors likely result from complex physiological interactions and could be an indicator of the pathophysiological effects occurring during immobilisation.

Understanding the cause of tremors is further complicated by our ability to quantify them. Tremor intensity is typically scored subjectively during the chemical immobilisation of wildlife (M. Hofmeyr, pers. comm., 2013), making it difficult to compare scores across studies or species. An objective method of measuring muscle tremor intensity, or at least validation of the visual scoring method, is needed to systematically investigate tremors. In the first part of our study, accelerometry, which has been used to measure hand tremors in humans (Elble et al. 2006), was used to quantify tremors in opioid-immobilised rhinoceroses, and we compared the tremor intensity measured by accelerometry to tremor scores recorded by a human observer.

To further understand the mechanisms underlying muscle tremors, the effects of various cardiorespiratory supportive interventions in etorphine–azaperone immobilised captive and free-ranging white rhinoceroses were evaluated (Haw et al. 2014, 2015). Tremor intensity and cardiorespiratory responses were evaluated and correlated after the administration of the following supportive interventions: oxygen insufflation, intravenous butorphanol, intravenous sterile water (control) or oxygen insufflation with intravenous butorphanol.

The aim of this study was to elucidate the mechanism for opioid-associated muscle tremors in white rhinoceroses and ascertain whether cardiorespiratory supportive interventions alter these tremors during immobilisation. Additionally, we aimed to validate methods for measuring muscle tremor.

## Research method and design

### Animals and chemical immobilisation

Our investigation formed part of a larger study that investigated pharmacological interventions aimed at improving the cardiorespiratory function of white rhinoceroses (*Ceratotherium simum*) during chemical immobilisation. The study was conducted in the Kruger National Park, South Africa (S24°59.696'E031°35.217, altitude 317 m) in two groups of rhinoceroses. One group consisted of eight sub-adult (3–5 years) boma-held male white rhinoceroses in which tremors were assessed during chemical immobilisation following four cardiorespiratory support interventions (Haw et al. 2014). The other group consisted of 14 free-ranging sub-adult (3–7 years) male white rhinoceroses in which tremors were studied during chemical immobilisation following a helicopter chase (Haw et al. 2015). Rhinoceroses were darted with a combination of etorphine hydrochloride (2.0 mg – 3.5 mg, M99, Novartis, Kempton Park, South Africa, 9.8 mg/mL), azaperone (30.0 mg – 52.5 mg, Stresnil, Janssen Pharmaceutical Ltd, Halfway House, South Africa, 40 mg/mL) and hyalase (2500 mg, hyaluronidase – lyophilised hyalase, Kyron Laboratories, Midrand, South Africa) by remote injection using a carbon dioxide (CO_2_)-powered dart gun (Dan-Inject, Skukuza, South Africa). The doses of the drugs were based on a sliding scale (Haw et al. 2014) that uses the known body mass of the animals (boma-held animals) or estimated body masses (free-ranging animals). Once immobilised, the animals were placed in lateral recumbency to reduce the risk of compression injuries in the limbs.

In the boma, 6 min into recumbency, each animal received one of four interventions randomly at 2-week intervals. These interventions included a control (sterile water, 2 mL, IV); continuous nasotracheal oxygen insufflation (30 L/min); butorphanol (15 × etorphine dose, IV, Kyron Laboratories, 20 mg/mL); and continuous nasotracheal oxygen insufflation (30 L/min) and butorphanol (15 × etorphine dose, IV) combined. In the interventions with oxygen insufflation, oxygen was administered continuously throughout the immobilisation using a nasogastric equine stomach tube (9.5 mm o.d. × 213 cm, Kyron Laboratories), which was placed into the trachea to the depth of the thoracic inlet. Butorphanol or sterile water was injected into an auricular vein. The free-ranging animals were immobilised only once, with the same immobilising drugs as in the boma studies, but received only butorphanol (15 × etorphine dose, IV) combined with continuous nasotracheal oxygen insufflation (30 L/min) as the supportive treatment.

At the end of each trial, the rhinoceroses were placed in a crate and weighed. Naltrexone, an opioid antagonist, was administered into an auricular vein (50 mg/mL, 20 × the etorphine dose, Kyron Laboratories) prior to the animal’s release.

### Measurement of tremor intensity

During chemical immobilisation, tremor intensities were subjectively scored by one observer using a pre-determined scale (Table 1) every 5 min in the boma-held animals and every minute in the free-ranging animals. In the free-ranging white rhinoceroses, a triaxial accelerometer data logger (Sigma-Delta logger, Mlog_AT1, Sigma-Delta Technologies, Perth, Australia) was also used to assess tremor intensity. This logger was attached to the lateral surface at the top of the upper forelimb and was set to measure activity counts every minute over 1-min intervals (epochs).

**TABLE 1:** Scoring system used to rank tremor intensity in the rhinoceros based on visual observations.

Observation	Score
No visible tremors	0
Slight tremors – tremors resulting in fine trembling of the legs and feet	1
Mild tremors – tremors resulting in fine trembling of the legs, feet, shoulder and chest	2
Moderate tremors – tremors resulting in gross trembling of the legs, feet shoulder and chest	3
Severe tremors – tremors resulting in gross trembling of the whole body and head	4

### Variables measured from blood

A medial auricular artery was catheterised using a 22 G × 25.4 mm IV catheter (Nipro Safelet Cath, Nipro Corporation, Bridgewater, NJ, USA). At 5, 10, 15 and 20 min (time 0 = start of lateral recumbency), 1 mL of blood was drawn into pre-heparinised syringes for blood gas analyses. In the boma-held animals, a portable pre-calibrated blood gas analyser with pre-calibrated blood gas cassettes (Roche OPTI CCA Analyzer + OPTI cassette B; Kat Medical, Johannesburg, South Africa) was used to determine the arterial partial pressure of oxygen (PaO_2_), carbon dioxide (PaCO_2_) and blood pH. In free-ranging animals, arterial blood samples were analysed using an Epoc^TM^ BGEM blood analysis system (Kyron Laboratories) to determine blood gases and pH, glucose, lactate, calcium (Ca^+^), chloride (Cl^-^), sodium (Na^++^) and potassium (K^+^) ion concentrations.

In the free-ranging animals, an 18 G × 25.4 mm IV catheter (Jelco^®^, Smiths Medical, Kempton Park, South Africa) was also placed into an auricular vein. Within 1 min after the rhinoceros was placed in lateral recumbency (time 0), a 10-mL blood sample was taken from this catheter. Venous samples were also drawn at 5, 10, 15 and 20 min and the blood was placed in two 5 mL EDTA (ethylene-diamine-tetra-acetic acid) tubes (BD Vacutainer^®^, Johannesburg, South Africa). These tubes were placed directly onto ice, and within 10 min of collection, they were centrifuged at 2500 rpm for 10 min. Plasma was aliquoted into cryotubes (Greiner Bio-One, Frickenhausen, Germany) and snap-frozen in liquid nitrogen. Samples were stored at -80 °C until catecholamine (noradrenaline and adrenaline) concentrations were determined using high-performance liquid chromatography (Agilent 1200 HPLC, Agilent Technologies, Santa Clara, CA, USA) using methods described by De Villiers et al. (1987) and Coetzee (2006).

### Statistical analysis

Repeated measures one-way analysis of variance (ANOVA) and a Friedman test were used to determine differences over time and between interventions at the different sampling intervals. A *post-hoc* Dunn’s test was used for non-parametric data (tremor intensity scores) and Tukey’s test for parametric data (PaO_2_, PaCO_2_, blood pH, glucose, lactate, electrolytes, and log-transformed tremor counts and catecholamine concentrations).

A Spearman’s rank-order correlation was used to determine the association between median tremor intensity scores and the mean PaO_2_, PaCO_2_ and blood pH over the interventions in the boma-held animals at 5, 10, 15 and 20 min. In the free-ranging animals, a Pearson’s product–moment correlation was used to determine the association between the mean of the variables over the immobilisation; non-parametric (tremor intensity counts, adrenaline and noradrenaline) data were log-transformed to the base of 10 before analysis. Median observed tremor intensity scores and median tremor intensity counts were correlated using Spearman’s rank-order correlation. Actual body mass, specific drug doses of etorphine and azaperone in mg/kg and total distance travelled were correlated (Spearman’s rank-order correlation) with tremor intensity counts at the 1- and 5-min time intervals. Data analysis was performed using GraphPad^®^ Prism (version 6.02) and *p* < 0.05 was considered significant.

## Results

### Tremors in immobilised free-ranging rhinoceroses

**FIGURE 1:**
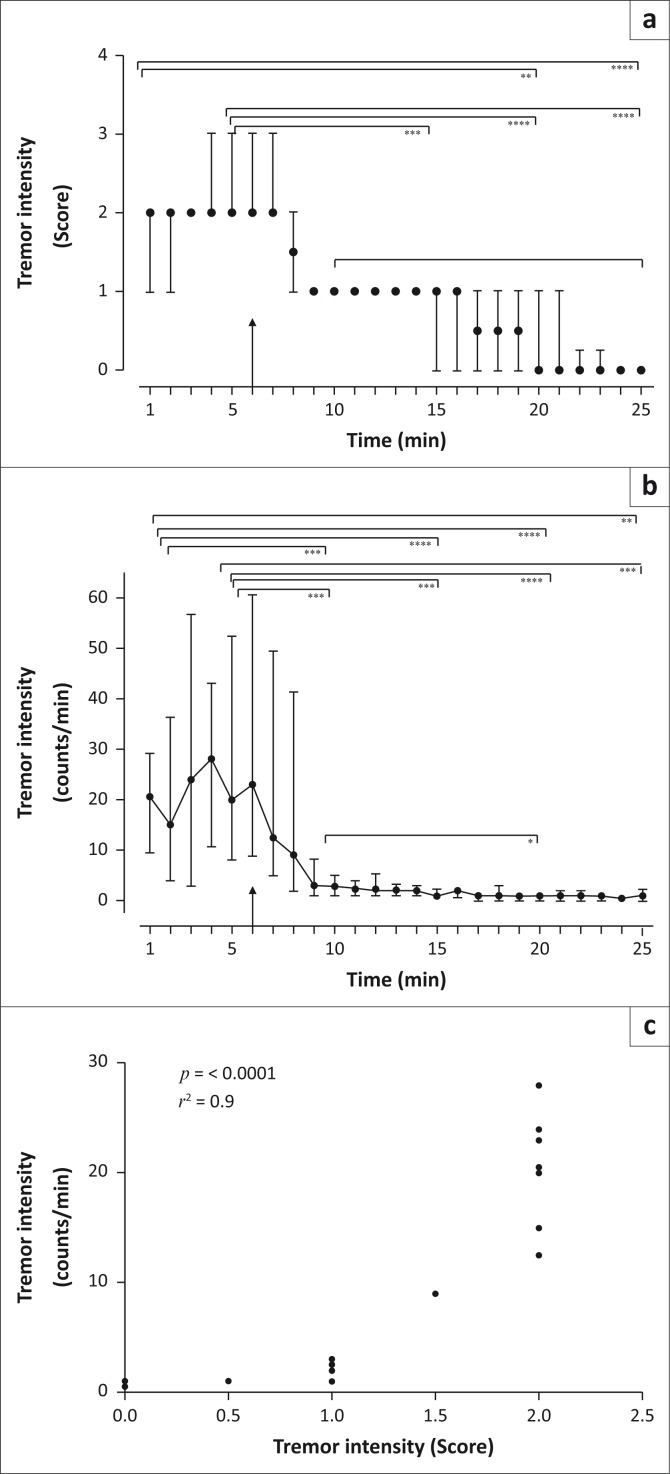
Median observed tremor intensity scores (a) and the median activity logger counts per minute (b), with interquartile ranges, of 14 free-ranging white rhinoceros that were chemically immobilised with a mixture of etorphine and azaperone. The arrow represents the time point at which butorphanol (IV) was administered and nasotracheal oxygen insufflation initiated. The brackets indicate which time intervals are significantly different from the other (*, *p* < 0.05; **, *p* < 0.01; ***, *p* < 0.001; ****, *p* < 0.0001; Friedman’s test with *post-hoc* Dunn’s tests for tremor intensity scores; ANOVA with *post-hoc* Tukey’s test on log-transformed tremor intensity counts; the data, however, are shown as actual counts). (c) Median tremor intensity (counts/min), which was recorded by the activity loggers, was compared to median tremor intensity (score), which was observed in the 14 white rhinoceros (Spearman rank-order correlation).

Tremors were at their greatest intensity immediately after the rhinoceros became immobilised (at time 1 and 5 min, Figure 1a, observed median tremor intensity score = 2; and Figure 1b, measured median tremor intensity = 20.0–20.5 counts per min). Tremor intensity decreased after the administration of butorphanol and commencement of oxygen insufflation (time 6–25 min, tremor intensity score = 0–3, *p* < 0.0001, *F* = 30.77, Figure 1a; and median tremor intensity = 1 counts per min, *p* < 0.0001, *F* = 27.68, Figure 1b).

Visual observations (subjective) and activity logger (objective) measurements reflected similar changes in the tremor intensities (Figure 1a and b) and were positively correlated (Figure 1c). The activity logger achieved a finer scale of measurement compared to the visual observations; therefore, associations between clinical variables and tremor intensity in the free-ranging animals were made using activity counts (counts per min) as a measure of tremor intensity.

### Relationship between tremor intensity and drug dose or exercise

In the free-ranging rhinoceroses, there was no correlation between total distance travelled from first sighting to immobilisation, and tremor intensity at 1 min (*p* = 0.47, *r*^2^ = 0.042) and 5 min (*p* = 0.88, *r*^2^ = 0.002). There was also no correlation between the dose of etorphine (median = 0.003 mg/kg, range = 0.003 mg/kg – 0.002 mg/kg) and azaperone (median = 0.034 mg/kg, range = 0.041 mg/kg – 0.028 mg/kg) and the tremor intensity at 1 min (*p* = 0.23, *r*^2^ = 0.12; *p* = 0.23, *r*^2^ = 0.12, respectively) or 5 min (*p* = 0.87, *r*^2^ = 0.003; *p* = 0.87, *r*^2^ = 0.003, respectively).

### Relationship between tremor intensity and partial pressure of oxygen, carbon dioxide or blood potential of hydrogen

**FIGURE 2:**
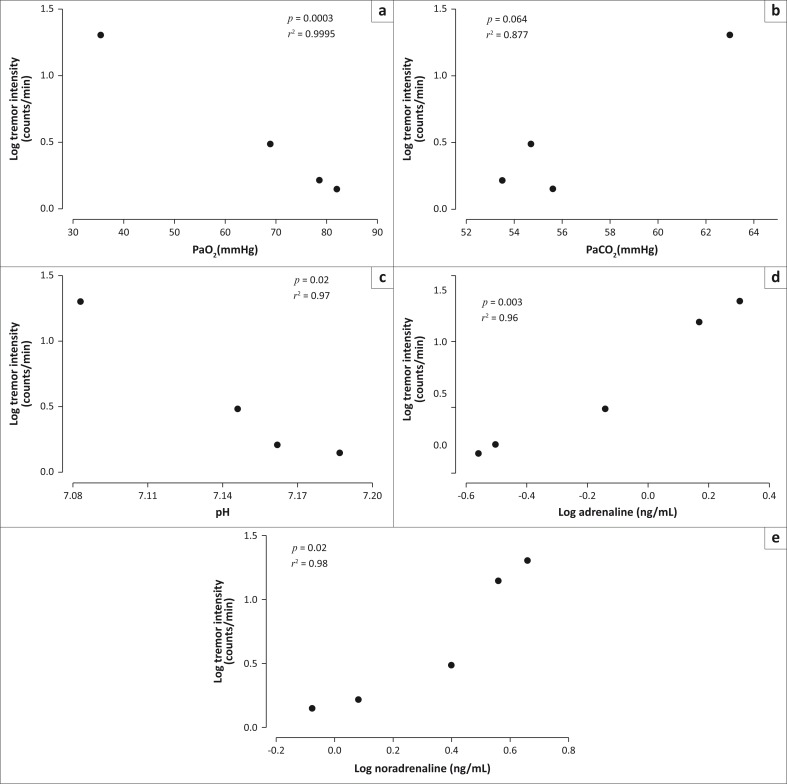
Fourteen free-ranging white rhinoceros were chemically immobilised with a combination of etorphine and azaperone. Six minutes after the animals were placed in lateral recumbency, they received a combination of butorphanol (IV) and nasotracheal oxygen insufflation. The log mean tremor intensity counts were correlated, using Pearson’s product–moment correlations, with the mean arterial partial pressure of oxygen (a) the mean arterial partial pressure of carbon dioxide (PaCO_2_), (b) and mean blood pH (c) (measured at 5, 10, 15, 20 and 25 min) and the mean log-transformed adrenaline (d) and noradrenaline (e) concentrations (measured at 1, 5, 10, 15, 20 and 25 min).

The immobilised free-ranging rhinoceroses were severely hypoxaemic (PaO_2_ = 35 mmHg ± 6.56 mmHg) at 5 min into the immobilisation. After the administration of butorphanol and oxygen insufflation, the PaO_2_ increased significantly over the rest of the immobilisation period (*p* < 0.0001, *F* >= 47.12; mean PaO_2_: 10 min = 69 mmHg ± 7.24 mmHg, 15 min = 79 mmHg ± 12.11 mmHg and 20 min = 82 mmHg ± 23.15 mmHg). PaO_2_ over the immobilisation period was negatively correlated with tremor intensity (Figure 2a).

These animals were hypercapnic (PaCO_2_ = 63 mmHg ± 7.70 mmHg) at 5 min post-immobilisation. After administration of butorphanol and oxygen insufflation, the PaCO_2_ decreased (*p* = 0.0002, *F* = 10.93; mean PaCO_2_: 10 min = 55 mmHg ± 8.47 mmHg, 15 min = 53.5 mmHg ± 6.88 mmHg and 20 min = 55.6 mmHg ± 4.93 mmHg). However, there was no relationship between PaCO_2_ and tremor intensity (Figure 2b).

These animals were also severely acidaemic 5 min into the immobilisation (mean pH = 7.08 ± 0.15). After the administration of butorphanol and oxygen insufflation, the blood pH increased (*p* < 0.0001, *F* = 28.97; mean pH: 10 min = 7.15 ± 0.14, 15 min = 7.16 ± 0.13 and 20 min = 7.19 ± 0.11). The blood pH was negatively correlated with tremor intensity (Figure 2c).

### Relationship between tremor intensity and plasma catecholamines

In the free-living rhinoceroses, plasma adrenaline concentrations were initially high (median adrenaline: 1 min = 1.23 ng/mL and 5 min = 1.58 ng/mL) but decreased significantly (*p* < 0.0001, *F* = 49.21) after the administration of butorphanol and oxygen insufflation (median adrenaline: 10 min = 0.56 ng/mL, 15 min = 0.2 ng/mL and 20 min = 0.2 ng/mL). Plasma adrenaline concentration was positively correlated with tremor intensity (Figure 2d).

Similarly, the plasma noradrenaline concentration was initially high (median noradrenaline: 1 min = 3.63 ng/mL and 5 min = 3.87 ng/mL) but decreased significantly (*p* < 0.0001, *F* = 50.51) after the administration of butorphanol and oxygen insufflation (median noradrenaline: 10 min = 1.91 ng/mL, 15 min = 0.89 ng/mL and 20 min = 0.72 ng/mL). The plasma noradrenaline concentration was positively correlated with tremor intensity (Figure 2e).

### Relationships between plasma catecholamines, arterial blood gases and potential of hydrogen

In the free-living rhinoceroses, mean log-transformed plasma adrenaline concentration was negatively and strongly correlated with mean PaO_2_ (*p* = 0.03, *r*^2^ = 0.95) and mean blood pH (*p* = 0.03, *r*^2^ = 0.94), but not with mean PaCO_2_ (*p* = 0.14, *r*^2^ = 0.75). The mean log-transformed plasma noradrenaline concentration was not correlated with mean PaO_2_ (*p* = 0.07, *r*^2^ = 0.86) or mean PaCO_2_ (*p* = 0.25, *r*^2^ = 0.57) but was correlated with mean blood pH (*p* = 0.04, *r*^2^ = 0.91).

### Relationships between tremor intensity and blood biochemical variables

Mean (± s.d.) values for blood biochemical variables for the immobilised white rhinoceroses before administration of butorphanol and oxygen insufflation were: pH 7.08 (±0.15), potassium: 4.1 (±0.4) mmol/L, sodium: 132 (±2) mmol/L, chloride: 99 (±3) mmol/L, calcium: 1.1 (±0.14) mmol/L, glucose: 8.8 (±1.8) mmol/L and lactate: 10.05 (±3.45) mmol/L. The blood concentration of potassium (K^+^) decreased significantly over the immobilisation period (*p* = 0.0014, *F* = 11.51), and it was the only electrolyte that was positively correlated with tremor intensity (*p* = 0.001, *r*^2^ = 0.997). Potassium was also negatively correlated with blood pH (*p* = 0.03, *r*^2^ = 0.95). Mean blood sodium (Na^+^, *p* = 0.93, *F* = 0.083), chloride (Cl^-^, *p* = 0.69, *F* = 0.071) and calcium (Ca^++^, *p* = 0.88, *F* = 0.44) concentrations did not change over the immobilisation period and were not correlated with log-transformed tremor intensity counts (Na^+^, *p* = 0.51, *r*^2^ = 0.24; Cl^-^, *p* = 0.45, *r*^2^ = 0.30; Ca^++^, *p* = 0.06, *r*^2^ = 0.89).

Blood glucose concentration increased significantly (*p* = 0.01, *F* = 6.12) over the immobilisation period, but was not correlated with the log-transformed tremor intensity count (*p* = 0.13, *r*^2^ = 0.76). Blood lactate concentration decreased over the immobilisation (*p* = 0.001, *F* = 15.60), but it was also not correlated with log-transformed tremor intensity count (*p* = 0.49, *r*^2^ = 26).

### Tremors in immobilised boma-held rhinoceroses

**FIGURE 3:**
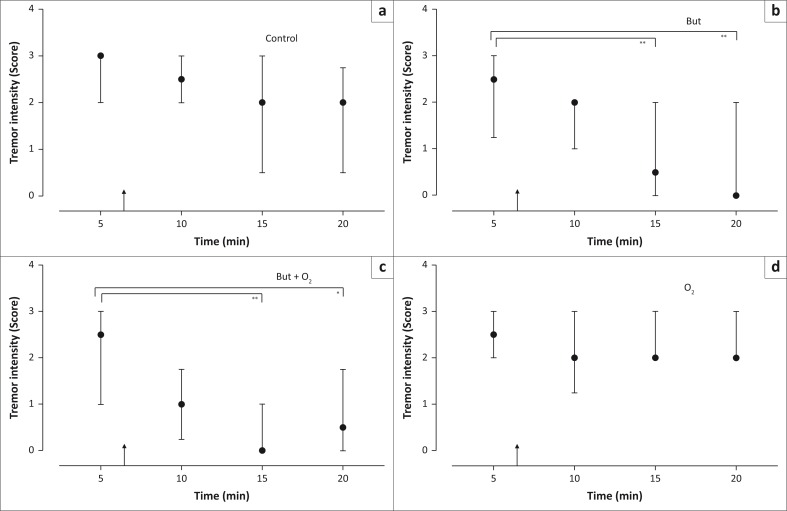
The median observed tremor intensity score with interquartile ranges for eight boma-held rhinoceros chemically immobilised with a mixture of etorphine and azaperone. Six minutes after they were placed into lateral recumbency, each rhinoceros received one of four interventions (arrow), on four separate occasions. The interventions included sterile water (IV) (control) (a), butorphanol (IV) only (b), the combination of butorphanol (IV) and nasotracheal oxygen insufflation (c), or nasotracheal oxygen insufflation only (d). Brackets indicate significant differences in tremor intensities compared to pre-intervention administration at 5 min (*, *p* < 0.05; **, *p* < 0.01; Friedman’s and post-hoc Dunn’s tests).

Tremor intensity was greatest at 5 min after the animals became recumbent (tremor intensity score = 2.5–3; Figure 3) and decreased over time during the immobilisation when the animals did not receive any supportive intervention (control trial, *p* = 0.003, *F* = 14.18; Figure 3a). However, the *post-hoc* Dunn’s multiple comparisons test revealed no significant changes in tremor intensity between measurement intervals (*p* = 0.1, 5 min vs. 10 min; *p* = 0.16, 5 min vs. 15 min and *p* = 0.06, 5 min vs. 20 min) in this control trial. After butorphanol was administered, tremor intensity decreased over time (*p* = 0.0003, *F* = 18.5; Figure 3b) to a median score of 0.5 at 15 min (*p* = 0.01, 5 min vs. 15 min) and 0 at 20 min (*p* = 0.003, 5 min vs. 20 min). Butorphanol combined with nasotracheal oxygen insufflation also decreased tremor intensity over time (*p* = 0.001, *F* = 17.02; Figure 3c) to a median score of 0 at 15 mins (*p* = 0.01, 5 min vs. 15 min) and 0.5 at 20 min (*p* = 0.02, 5 min vs. 20 min). In contrast, when nasotracheal oxygen insufflation was administered on its own, there was no change in tremor intensity over time (*p* = 0.06; *F* = 7.44, Figure 3d) during the immobilisation.

Tremor intensity at 5 min was not different amongst the four trials (*p* = 0.28, *F* = 3.84; Figure 3). Although there was a significant difference in the tremor intensity between the interventions at 15 min (*p* = 0.0014, *F* = 15.63) and 20 min (*p* = 0.0022, *F* = 14.56), the *post-hoc* Dunn’s multiple comparisons test did not identify where the differences occurred. However, at 10 min (*p* = 0.012, *F* = 10.500), tremor intensity was lower in the animals receiving butorphanol and oxygen compared with those in the control group (*p* = 0.02; score 2.5 vs. 1, Figure 3a vs. 3c).

In all the trials at 5 min, the animals were severely hypoxaemic (PaO_2_ < 35 mmHg), hypercapnic (PaCO_2_ > 80 mmHg) and acidaemic (pH < 7.3), and there was no difference in the PaO_2_ (*p* = 0.16, *F* = 2.12), PaCO_2_ (*p* = 0.6, *F* = 0.58) and pH (*p* = 0.43, *F* = 0.897) between trials. The effect of the supportive interventions on blood gas variables has been described by Haw et al. (2014). Tremor intensity score was negatively correlated with PaO_2_ at 5 min (*p* < 0.0001, *r*^2^ = 0.6), 10 min (*p* < 0.0001, *r*^2^ = 0.9) and 15 min (*p* < 0.0001, *r*^2^ = 0.9) over the trials. There was also a negative correlation between the tremor intensity score and the blood pH over the trials, but only at 20 min (*p* < 0.0001, *r*^2^ = 0.9). However, there was no correlation between tremor intensity score and PaCO_2_ over the trials at any of the time points (5 min, *p* = 0.5, *r*^2^ = 0.77; 10 min, *p* ³ 0.99, *r*^2^ = 0.32; 15 min, *p* = 0.33, *r*^2^ = 0.74 and 20 min, *p* = 0.17, *r*^2^ = 0.95).

## Ethical considerations

The study was approved by the Animal Use and Care Committee of SANParks (HAWA1042), the Animal Ethics Screening Committee of the University of the Witwatersrand (clearance 2012/23/04) and the University of Pretoria (approval number V087/13).

## Discussion

White rhinoceroses, immobilised with etorphine and azaperone, exhibited muscle tremors irrespective of whether they were in a boma or field setting. The intensity of these tremors was not associated with the dose of the capture drugs used in this study or distance the animals travelled before they became recumbent in the field. Tremors lasted for 20 min after recumbency in animals that did not receive butorphanol with or without oxygen. We validated the visual observation tremor scores by comparing them to leg motion detected by accelerometry. Tremor intensity was measured to a finer scale by the accelerometer logger compared to visual observations and, therefore, we used the activity counts measured by the logger in the field immobilised rhinoceroses to determine associations between tremor intensity and other measured physiological variables.

We found that the greatest tremor intensity occurred when the animals were severely hypoxaemic and acidaemic. In field immobilised rhinoceroses, tremor intensity was negatively correlated with arterial blood PaO_2_ and pH, but had no relationship with PaCO_2_ or lactate. Tremor intensity was also positively correlated with plasma catecholamine concentrations. These catecholamine concentrations in turn were negatively correlated with arterial blood PaO_2_ and pH. These findings suggest that hypoxaemia and acidaemia may have been associated with tremor intensity by stimulating the release of catecholamines. The administration of butorphanol plus oxygen lessened the hypoxaemia and acidosis, with concurrent decreases in catecholamine and tremor intensities.

Tremors have previously been reported in white rhinoceroses that were immobilised with etorphine and azaperone (Atkinson et al. 2002; Fahlman 2008; Moreira 2010; Portas 2004; Radcliffe et al. 2000). Indeed, the Rhinocerotidae and Equidae, both of the order Perissodactyla, appear to be particularly prone to developing muscle tremors following opioid administration (Haigh 1990; Moreira 2010). Etorphine is a potent opioid agonist at all the opioid receptors (Haigh 1990). Muscle tremors have been described during etorphine-induced immobilisation in other mammalian species (Bush et al. 2004; Haigh 1990; Mentaberre et al. 2010; Moreira 2010; Portas 2004), but the mechanism causing this effect has not yet been elucidated.

We found no correlation between etorphine doses used in this study and the tremor intensity in the rhinoceroses and therefore concluded that tremor intensity is not a simple dose-dependent drug effect. However, our study was not designed to test the specific effects of drugs on tremors. Therefore, further pharmacological studies are needed to adequately assess the role that opioid-induced receptor activation or deactivation may play in causing tremors. That butorphanol, an opioid agonist–antagonist, reduced tremor intensity in this and other studies (Burroughs et al. 2012b; Moreira 2010) suggests that a direct pharmacological mechanism may be involved in the cause of tremors. However, butorphanol is administered during chemical immobilisation to correct hypoxaemia, hypercapnia and acidosis associated with etorphine-induced respiratory compromise. Because these physiological derangements may influence tremors, it is plausible that butorphanol’s effects on tremors may also be caused indirectly by alleviating etorphine-induced respiratory compromise. In the boma-managed white rhinoceroses, we found a decrease in the tremor intensity after butorphanol was administered, but this decrease was no different compared to when the animals received sterile water in the control trial. However, when oxygen insufflation was combined with butorphanol, the decrease in observed tremor intensity was greater than that measured in the control trial. This combination corrected the hypoxaemia, but butorphanol alone only partially reduced hypoxaemia in these animals (Haw et al. 2014). Furthermore, across the groups, there was a strong correlation between tremor intensity and PaO_2_, suggesting that the physiological alterations induced by butorphanol combined with oxygen insufflation played a role in altering tremor intensity in these animals.

In the field immobilised animals that received butorphanol with oxygen insufflation, there was a strong negative correlation between tremor intensity and pH, and PaO_2_. A decrease in pH is normally associated with a decrease in force and contractility of skeletal muscle (Stackhouse, Reisman & Binder-Macleod 2001). Similarly, hypoxia usually reduces muscle contractility and causes weakness (Berne & Levy 2000). Tremors are normally associated with increased tension and muscle rigidity; therefore, it is unlikely that hypoxia and acidaemia caused tremors by their direct effects on the muscles of the rhinoceros.

A plausible explanation for the relationship between muscle tremors and hypoxia, as well as acidaemia, is chemoreceptor activation of the sympathetic nervous system, which would increase the release of tremor-inducing catecholamines, in particular, adrenaline (Berne & Levy 2000; Prabhakar 2000). In the rhinoceros, PaO_2_ and pH were negatively correlated with adrenaline, and all three variables were correlated with tremor intensity. An increase in plasma adrenaline concentration during hypoxaemia in mammals is well described (Perry et al. 1989; Rose et al. 1977; Wasser & Jackson 1991; Yates et al. 2012). In addition, both a direct activation of the adrenal gland (Nahas 1970) and indirect activation of the sympathetic nervous system (Jeffers 1986) associated with increasing hydrogen ion concentrations during acidosis result in an increase in secretion of adrenaline. Considering the effects of low pH, and the consequent release of adrenaline, we expected that PaCO_2_ and blood lactate would have also been correlated with tremor intensity, but they were not. We surmise that the summative effects of these two hydrogen ion–forming substances played a greater role in influencing adrenaline release and tremor intensity than did either substance on its own.

Adrenaline is known to be tremorgenic predominantly through activation of β_2_-adrenoreceptors in skeletal muscles (Foley, Marsden & Owen 1967; Lakie 2010; Marsden & Meadows 1970), and increased blood catecholamine concentrations have been associated with increased tremor intensity (Cazzola & Matera 2012; Frishman 2003; Marshall & Schnieden 1966). In order for adrenaline to cause tremors, its concentration in blood needs to exceed that of normal circulating physiological concentrations. As far as we are aware, there are no documented normal values for blood catecholamine concentrations in rhinoceroses measured at rest. Since the horse is related to the rhinoceros, we compared catecholamine concentrations in this species to that of our rhinoceroses. Plasma adrenaline (1.65 ng/mL ± 5.03 ng/mL) and noradrenaline (3.53 ng/mL ± 5.03 ng/mL) concentrations in the immobilised rhinoceroses at 1 and 5 min after the animals became recumbent in the field were higher than those recorded from horses at rest (normal plasma adrenaline – 0.055 ng/mL ± 0.005 ng/mL and normal plasma noradrenaline – 0.124 ng/mL ± 0.011 ng/mL; Nagata et al. 1999). The initial catecholamine concentrations after the animals became recumbent were also most likely influenced by the stress of capture (Burroughs et al. 2012b) and, potentially, by the sympathomimetic effects of etorphine (Daniel & Ling 1972). Based on our study design, we were not able to distinguish these effects on catecholamine concentrations from those of hypoxaemia and acidaemia. However, irrespective of the cause of catecholamine increase and tremors, we have shown that the correction of hypoxaemia and acidaemia played a substantial role in reducing catecholamine concentrations and tremor intensity.

Changes in other physiological variables, such as electrolytes and glucose, may also result in a change in tremor intensity (Carithers 1995). Low blood calcium has been implicated in causing tremors (Carithers 1995). However, the immobilised rhinoceroses in the field were not hypocalcaemic (Miller & Buss 2012), and there was no relationship between blood calcium and tremor intensity. Blood sodium ion concentrations did not change over the immobilisation period and were similar to those previously reported as normal for white rhinoceros (Miller & Buss 2012). Blood chloride ion concentrations, which also did not change over the immobilisation period or correlate with tremor intensity, were slightly higher in our study compared with those reported as normal values (Miller & Buss 2012). Potassium was the only electrolyte that had a significant positive correlation with tremor intensity. However, this relationship may simply have been a reflection of the effect of the changes in blood pH, as blood pH strongly influences the movement of potassium in and out of the extracellular space and, hence, impacts blood potassium concentrations (Burnell et al. 1956). Additionally, hyperkalaemia, but not hypokalaemia, is known to cause muscle tremors (Carithers 1995). In our rhinoceroses, blood potassium ion concentrations were lower than those reported as normal for white rhinoceroses (Miller & Buss 2012). Hypoglycaemia has also been implicated in causing tremors (Carithers 1995), but there was no relationship between the blood glucose concentration and the tremor intensity in the field-captured animals.

Therefore, the tremors that occur during etorphine–azaperone immobilisation are strongly associated with physiological responses induced mainly by hypoxaemia and acidaemia, but potentially also influenced by the psychological stress response associated with capture and etorphine-induced catecholamine release. These tremors mimic trembling that occurs in humans that are exposed to a severe physiological stress or a frightful situation (Findley 1996) and therefore could be similarly termed stress-induced trembling.

## Limitations of the study

This study has shown that there are multiple variables that need to be taken into consideration when investigating tremor intensity in rhinoceroses during chemical immobilisation. It would have been ideal to use the activity loggers in the boma study to better clarify the effects of the drugs on muscle tremors. However, due to a different pre-determined study design, the use of respective measurement equipment was not feasible.

## Conclusion

When white rhinoceroses were immobilised with etorphine–azaperone, they developed tremors. These tremors were associated with pathophysiological effects of the immobilising drugs, which caused the release of tremorgenic catecholamines. The pathophysiological effects most strongly associated with tremors were hypoxaemia and acidaemia. The administration of the combination of butorphanol and nasotracheal oxygen insufflation corrected hypoxaemia and improved acidaemia, which was associated with a reduction in catecholamine concentrations, and reduced or abolished tremors during immobilisation. Therefore, tremors can be treated effectively in etorphine–azaperone immobilised rhinoceroses if an animal’s blood oxygen levels and pH are corrected using butorphanol and nasotracheal oxygen insufflation. Lastly, tremor intensity could possibly be used clinically to indicate the extent of an animal’s respiratory and metabolic compromise, and the extent of other catecholamine related side-effects, and could thus be used as an early warning signal to help reduce the risks associated with rhinoceros immobilisation.
